# Global prevalence of advanced HIV disease in healthcare settings: a rapid review

**DOI:** 10.1002/jia2.26415

**Published:** 2025-02-06

**Authors:** Nathan Ford, Reshma Kassanjee, Dominik Stelzle, Joseph N Jarvis, Omar Sued, Georges Perrin, Meg Doherty, Ajay Rangaraj

**Affiliations:** ^1^ Global HIV, Hepatitis and STIs Programs World Health Organization Geneva Switzerland; ^2^ Centre for Integrated Data and Epidemiological Research School of Public Health University of Cape Town Cape Town South Africa; ^3^ Department of Clinical Research, Infectious and Tropical Diseases London School of Hygiene and Tropical Medicine London UK; ^4^ Botswana Harvard Health Partnership Gaborone Botswana; ^5^ HIV, Hepatitis, Tuberculosis, and Sexually Transmitted Infections Unit Pan American Health Organization Washington DC USA; ^6^ HIV, Tuberculosis, Hepatitis and STIs (HTH) Unit World Health Organization Brazzaville Republic of the Congo

**Keywords:** advanced HIV disease, CD4 cell count, disengagement, hospital, healthcare setting, severe illness

## Abstract

**Introduction:**

Recent studies have indicated a high enduring burden of advanced HIV disease, but estimates across regions and settings are lacking. The aim of this study was to estimate the prevalence of advanced HIV disease since 2015 among those people with CD4 measured in healthcare settings, disaggregated by age group, level of healthcare and region.

**Methods:**

We searched MedLine via Pubmed and Hinari for studies that reported the proportion of individuals with advanced HIV disease (defined as CD4 cell count <200 cells/mm^3^) in healthcare settings since 2015; this search was complemented by conference abstracts and data from the International epidemiology Databases to Evaluate AIDS Consortium (IeDEA). We estimated pooled prevalence of advanced HIV disease using random‐effects models and performed subgroup and sensitivity analyses to explore heterogeneity.

**Results:**

We obtained data from 117 cohorts, representing 1,814,362 individuals from 52 countries across all six World Health Organization regions. The majority of studies (*n* = 83) were conducted among adults and recorded CD4 cell count among treatment naïve individuals at antiretroviral therapy start (*n* = 86). Studies included data reported up to 2023. The proportion of individuals with advanced HIV disease was higher in inpatient settings (44.3%, 95% CI 39.1−49.6%) compared to outpatient settings (33.5%, 95% CI 31.5−35.4%). Prevalence was similar across age groups, time since HIV diagnosis and treatment status, and highest in West and Central Africa, South‐East Asia and the Eastern Mediterranean region.

**Discussion:**

This review finds that at least a third of people presenting to healthcare settings have advanced HIV disease, with no evidence that this has changed in recent years. Screening for advanced HIV remains important to be able to direct appropriate preventive, diagnostic and therapeutic interventions to prevent progression to severe illness and death.

**Conclusions:**

This review summarizes recent evidence of the continued high proportion of individuals who (re)present to care with advanced HIV disease. These findings underscore the urgent need to reinforce programme capacity to diagnose, prevent and treat advanced HIV disease as an essential pillar of the global AIDS response.

## 1 INTRODUCTION

Since 2015, the World Health Organization (WHO) recommended providing antiretroviral therapy (ART) to all people living with HIV irrespective of CD4 cell count. This *Treat All* recommendation has been adopted as a national policy worldwide, and has reduced HIV transmission and mortality, and increased the life expectancy of individuals living with HIV [1]. Notwithstanding the considerable progress in increasing access to HIV testing and treatment over the last decade, advanced HIV disease remains an important challenge, contributing to a high ongoing burden of severe opportunistic infection, hospitalization and death.

Timely identification of advanced HIV disease with CD4 testing is an important programmatic activity. However, CD4 testing has declined in many low‐ and middle‐income settings since the *Treat All* recommendation was established, and with this the ability to identify and report the number of individuals with advanced HIV disease. Recent studies from high [2] and low‐income [3] settings that do report CD4 cell count have indicated a high enduring burden of advanced HIV disease. This is in part due to late presentation to care and delayed ART initiation, but increasingly because of individuals cycling out of treatment, returning after a period of treatment interruption [4, 5].

We undertook a rapid review to estimate the prevalence of advanced HIV disease among individuals receiving care in both inpatient and outpatient settings who had a CD4 cell count measurement. This review focused on data published since 2015, the year when the *Treat All* recommendation was established.

## 2 METHODS

### 2.1 Study and data sources

The study was conducted according to PRISMA guidelines following a protocol registered in PROSPERO (CRD42024537211). We screened MedLine via Pubmed and Hinari to search for studies that included the term “advanced HIV disease” (defined as CD4 cell count <200 cells/mm^3^) in the title or abstract. We also screened abstracts of the International AIDS Society Conference and the Conference on Retroviruses and Opportunistic Infections from 2022 onwards to identify recent studies that have not yet been published in full. We included all studies which reported data between 1st January 2015 (the start of “Treat All”) and 01 March 2024. No language or geographical restrictions were applied. Additional data were includeds from a national HIV programme managers meeting in Thailand in 2024 [6].

We also included country‐level aggregate observational data from HIV treatment programmes provided by the International epidemiology Databases to Evaluate AIDS Consortium (IeDEA) on proportions of persons with CD4 cell count <200 cells/mm^3^ at ART start at collaborating treatment programmes, stratified by child and adult populations [7]. Reflecting general trends, the proportion of persons in whom CD4 cell count is measured at ART start within IeDEA programmes has declined substantially for countries in sub‐Saharan Africa since 2015: in 2019, over 80% of persons living with HIV in IeDEA programmes in sub‐Saharan Africa (except South Africa) did not have a CD4 test; these estimates, therefore, relate to the small proportions of persons in whom CD4 is measured [8].

### 2.2 Study inclusion and data extraction

Studies were eligible if they included at least 20 participants within a routine healthcare setting; as such, clinical trials and household surveys were excluded. Studies which spanned periods before and after 01 January 2015 were included if it was possible to disaggregate data from 2015 onwards. We included individuals presenting to care for any reason. Data were disaggregated according to inpatient or outpatient setting, with inpatient settings defined by the studies as a hospital admission. Studies which included a mix of patients were classified according to the majority (>50%) and where such data on the setting were not available, the study was reported as inpatient/outpatient in subgroup analyses. Studies were excluded if they reported exclusively on cohorts of patients living with HIV and other diseases. To assess study quality, we used items of the JBI tool to assess participant recruitment, data completeness, method of data measurement and outcome ascertainment; this information was used to inform the evidence certainty assessment (Supplementary Appendix) [9]. Evidence certainty was assessed using the GRADE framework. For the purposes of this review, we defined a child as aged <15 years.

Data extraction was conducted by one author (NF) and verified by a second author (AR, DS) using a predefined data extraction sheet. If outcomes from the same cohort were published across different publications, each outcome was only reported once.

### 2.3 Data analysis

We calculated proportions and corresponding 95% confidence intervals (CIs) for the proportion of individuals with advanced HIV disease and pooled data after transformation using random‐effects meta‐analysis [10, 11]. Outcomes of interest were the proportion of patients with advanced HIV disease within a defined cohort. We performed subgroup analyses to assess differences in the proportion of individuals with advanced HIV disease by location (WHO region and income group), age, ART status, new or previous HIV diagnosis and date of data extraction (before or after 2020, to evaluate differences between older and more recent studies). A full linear time‐trend analysis was not possible because individual studies reported data over varying time periods; however, where individual studies provided data across time, this information was highlighted. These pairwise subgroup proportions were limited to outpatient and mixed (i.e. inpatient and outpatient) settings with inpatient studies dropped from these analyses to ensure comparability, and the limited number of inpatient studies prevented separate analyses for this group; subgroups were compared using a two‐sample *z*‐test for two groups. Statistical tests for heterogeneity do not work well with pooled proportions [12], thus we assessed sources of heterogeneity through visual inspection of forest plot, exploration of outliers and the influence of larger studies on overall estimates [13]. We analysed all data with Stata (version 15.0).

### 2.4 Role of the funding source

The funder of the study had no role in study design, data interpretation or writing of the report.

## 3 RESULTS

**Figure 1:**
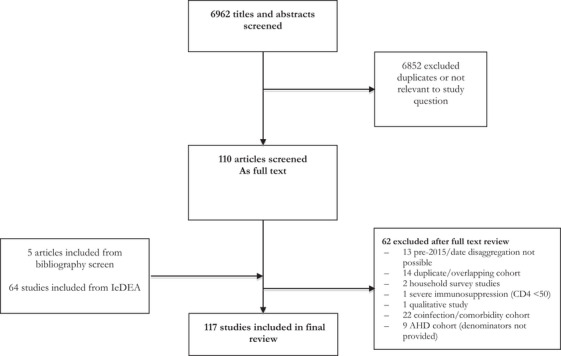
Study selection process.

From an initial screen of 6962 reports, 110 studies were screened as full text and 53 studies were taken through to review (Table 1). An additional 64 study estimates from 38 countries were provided by IeDEA. Together, these 117 studies provided data on 1,814,362 individuals from 53 countries across all WHO regions (Figure 1). The majority of data were from the AFRO region (1,570,490 individuals), including 20 countries (1,547,559 individuals) from eastern and southern Africa and 11 countries (22,931 individuals) from West and Central Africa. Data from 151,155 individuals from 38 countries was contributed by IeDEA. Most studies were conducted among adults (81 studies) and recorded CD4 cell count among individuals at ART start (86 studies). Studies reported data up to 2023, with almost a third (30%, 557,824 individuals) reporting data from 2020 onwards. Fifty‐one cohorts were from outpatient settings, eight from inpatient settings and the remainder (including most IeDEA sites) were from diverse sites. Studies included individuals who were newly diagnosed (28 studies) and those who had been diagnosed previously (42 studies).

**Table 1:** Study characteristics

Study	Country	Date of data extraction	Population	Newly diagnosed	Data source	Sample size	Number with advanced HIV disease
Afrashteh et al. [14]	Iran	2018−2021	Adults	No	Counselling centre register	249	163
Alli et al. [15]	Malawi	2022	NS	Yes	Hospital records	679	288
Baldeh et al. [16]	Sierra Leone	2022−2023	Adults	No	Hospital records	231	35
Benzekri et al. [17]	Senegal	2017−2018	Adults	No	HIV testing and treatment sites	185	102
Bwalya et al. [18]	Zambia	2022	NS	No	Hospital records	70	30
Chabikuli et al. [19]	DRC	2015−2020	Adults, adolescents and children	No	Health facility data	12,699	5537
Daama et al. [20]	Uganda	2021−2022	Adults, adolescents and children	Yes	Health facility EMR	2254	518
Dat et al. [21]^a^	Vietnam	2015−2017	Adults	Yes	Outpatient clinical records	3504	1354
Ditondo et al. [22]	DRC	2018−2022	Adults	No	Health facility data	573	288
Elgalib et al. [23]	Oman	2015−2019	Adults	Yes	National HIV surveillance system	603	279
Elizalde‐Barrera and Juarez‐Mendoza [24]	Mexico	2015−2021	Adults	Yes	Newly diagnosed PLHIV referred to care	348	158
Garcia‐Ruiz De Morales et al. [25]	Spain	2017−2022	Adults	Yes	Primary care centres	5200	1185
Gimenez‐Arufe et al. [26]	Spain	2015−2020	Adults	Yes	Newly diagnosed PLHIV, EMR	167	55
Glencross et al. [27]	South Africa	2015	Adults	No	Health facility data	8239	2200
Hamzah et al. [28]	UK	2022	Adults	Yes	Outpatient HIV testing in emergency department	128	53
Heller et al. [29]	Malawi	2020	Adults	No	Medical ward data	460	245
Huang et al. [30]	China	2018−2021	Adults	Yes	Medical records	600	232
Jiang et al. [31]	China	2018−2021	Adults	Yes	National HIV surveillance system	997	400
Kerschberger et al. [32]	Eswatini	2016	Adults	Yes	National ART treatment database	1888	620
Kumar and Singh [33]	India	2017	Adults	No	ART centre records	84	40
Lamp et al. [34]	Cameroon, Mozambique, Zimbabwe, Uganda	2016	NS	No	Routine testing data captured by PIMA analyser	639,658	102,984
Lauscher et al. [2]	Germany	2019−2020	Adults	Yes	Health centre data	706	236
Leeme et al. [3]	Botswana	2015−2017	Adults	Yes	HIV reference laboratory data	14,423	3571
Levy‐Braide et al. [35]	Nigeria	2021−2022	NS	Yes	Facility records	11,781	5487
Li [62]	China	2016−2020	Adults	Yes	Comprehensive Response Information Management System	8575	4027
Lifson et al. [36]	Ethiopia	2015−2017	Adults	No	Records from 16 district hospitals and 16 health centres	1559	942
Mambetov et al. [37]	Kyrgyzstan	2020−2021	NS	Yes	National database	240	79
Masaba et al. [38]	Kenya	2016−2019	Adults	Yes	EMR and paper‐based records	19,427	6649
Mayasi Ngongo et al. [39]	DRC	2017−2020	Adults	No	Monitoring data from 84 health facilities	7908	
Mugenyi et al. [40]	Uganda	2017−2022	Adults	Yes	Clinic data	10,446	2151
Musengimana et al. [41]	Rwanda	2016−2018	Adults	No	EMR	957	105
Mwakisambwe et al. [42]	Tanzania	2020−2021	Adults	Yes	Routine healthcare data	70,302	13,994
Nalintya et al. [43]	Uganda	2019−2022	NS	No	Routine clinic data	39,903	6171
Ndlovu et al. [44]	Malawi, Zimbabwe, DRC	2017	Adults	No	Clinic data	708	221
Nhampossa et al. [45]	Mozambique	2015−2020	Adults	No	Clinic data	2458	349
Noknoy [6]	Thailand	2015−2022	Adults, adolescents and children	No	Clinic data	149,521	50,673
Oboho et al. [46]	Uganda, Kenya, Tanzania, Nigeria	2015−2021	Adults	No	Clinic data	23,288	1919
Osler et al. [4]	South Africa	2015−2017	Adults	No	Data linkage between lab reported CD4 and HIV treatment information systems	557,566	116,234
Otani et al. [47]	Japan	2015−2019	Adults	Yes	>100 collaborating institutions of a drug resistance HIV surveillance network	2533	1141
Ousley et al. [48]	Kenya and DRC	2015−2017	Adults	No	Clinic data	707	460
Owachi et al. [49]	Uganda	2020−2023	Adults	No	Hospital data	5827	2271
Parisi et al. [50]	USA	2015−2021	Adults	Yes	Enhanced HIV/AIDS Reporting System	27,460	6316
Pedrola et al. [51]	Argentina	2022	Adults, adolescents and children	Yes	Database of 35 testing centres	620	129
Raberahona et al. [52]	Madagascar	2015−2016	Adults	Yes	Inpatient and outpatient medical records	150	90
Samayoa et al. [53]	Guatemala	2017	Adults	Yes	13 HIV clinics	1323	630
Shi et al. [54]	China	2015−2020	Adults	Yes	Provincial surveillance system	20,791	14,572
Spinelli et al. [55]	USA	2021	Adults	No	Clinic data	1816	272
Ssempijja et al. [56]	Uganda	2018−2020	Adults	No		187	48
Stoger et al. [57]	Tanzania	2016−2019	Adults	Yes	Clinic data	1046	583
Subramanian et al. [58]	India	2022	Adults	—	Clinic data	1504	255
Dagnaw Tegegne et al. [59]	Ethiopia	2015−2020	Adults	No	Hospital data	354	115
Tiam et al. [60]	Lesotho	2018−2019	Adults	No	Clinic data	150	70
Yendewa et al. [61]	Sierra Leone	2017	Adults	Yes	Hospital data	155	76

Abbreviations: EMR, electronic medical record; NS, not stated; PLHIV, people living with HIV.

^a^ Study limited to individuals with CD4 <100.

Overall, studies were rated as being at moderate risk of bias (Supplementary Appendix), with considerable heterogeneity in outcomes, as expected considering differences in healthcare setting, national HIV epidemic and programmatic response. The certainty of the evidence was rated as low due to concerns related to risk of bias (representativeness of the study population and retrospective data collection), and imprecision and inconsistency in the subgroup estimates (notably setting and region).

**Figure 2:**
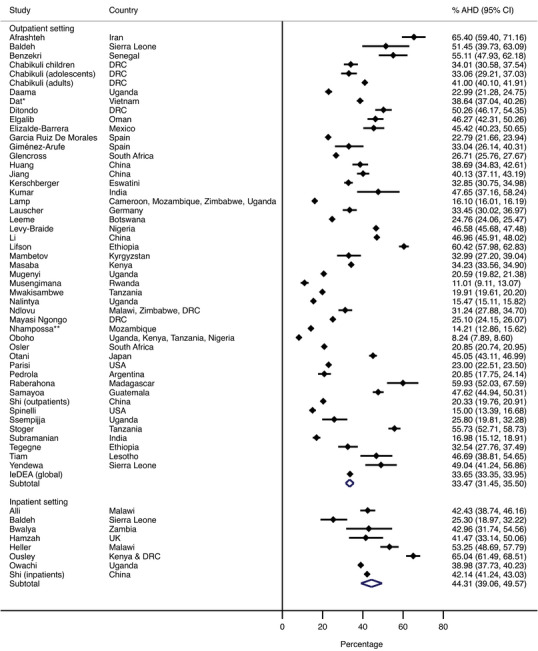
Summary estimates of advanced HIV disease prevalence in health care settings. AHD, advanced HIV disease. Studies that reported data from both inpatient and outpatient settings not included.

The pooled proportion of advanced HIV disease was higher in inpatient settings (44.3%, 95% CI 39.1−49.6%) compared to outpatient settings (33.5%, 95% CI 31.5−35.4%) (Figure 2). Two studies reported data stratified by healthcare setting: the first study used data from a provincial surveillance system in China and found a higher prevalence of advanced HIV disease among inpatients (42.1%, 41.2−43.0%) compared to outpatients (20.3%, 19.8−20.9%) [54]; the second study, using data from a tertiary hospital in Sierra Leone, found a higher prevalence of advanced HIV disease among inpatients (51.5%, 39.0−63.8) compared to outpatients (39.3%, 31.7−47.2), but this difference was not significant (*p* = 0.1).

**Figure 3:**
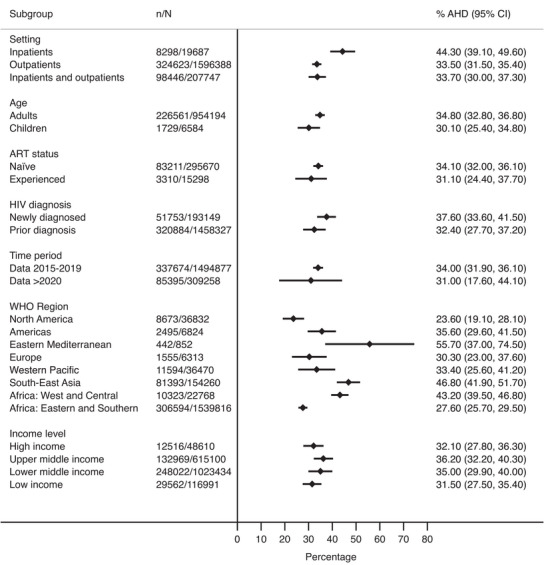
Subgroup analyses. AHD, advanced HIV disease; ART, antiretroviral therapy. All analyses other than setting restricted to outpatient and mixed settings (inpatient studies dropped from analysis).

In prespecified subgroup analyses for outpatient and mixed settings, overall estimates were similar across age groups, comparing studies limited to ART naïve individuals and studies in which the majority of participants (>50%) reported taking ART, and time since HIV diagnosis (newly diagnosed vs. previously diagnosed). Prevalence was similar comparing studies that only included data from 2020 onwards (31.0%, 95% CI 17.6−44.1%) and studies reporting data from 2015 to 2019 (34.0%, 95% CI 31.9−36.1%), consistent with studies which reported data over time [3, 6, 23, 26, 34, 54, 62]. Prevalence was highest in West and Central Africa, South‐East Asia, the America and the eastern Mediterranean region (Figure 3). There was no difference by country economic grouping.

In a sensitivity analysis assessing the possible influence of larger studies, the overall estimate did not change importantly if the three largest studies (each with *n*>100,000) [4, 6, 34] or if data from IeDEA were excluded from the analysis (both *p* = 0.8).

Seven studies provided near complete datasets for CD4 cell counts for a given region: a national surveillance system of Oman [23], electronic medical records for a health catchment area in Spain [26], a centralized database of all CD4 measures transmitted via the Alere Pima Analyzer in Cameroon, Mozambique, Uganda and Zimbabwe [34], a central reference laboratory processing nearly all CD4 measures in Gaborone, Botswana [3], a case reporting system in southwest China [62], a provincial dataset from eastern China [54] and a national surveillance system in Thailand [6]; a number of these studies provide estimates of trends over time [6, 24, 26, 34, 54, 62]. No difference was found comparing prevalence estimates derived from these datasets and the other studies in these regions.

## 4 DISCUSSION

This review provides further evidence that, despite good progress towards meeting the 95‐95‐95 global targets and improved access to diagnosis and ART, advanced HIV disease remains an important challenge across age groups. We found that around a third of individuals initiating ART in healthcare settings and in whom CD4 is measured have advanced HIV disease, with a higher proportion among PLHIV admitted to hospital.

These findings confirm the importance of maintaining a focus on reducing HIV‐associated illness and death as a core component of the global AIDS response. An essential first step is to ensure that programmes can and do screen correctly for advanced HIV disease among the following: individuals who present to care; those who re‐present after a period of disengagement; those found to have non‐suppressed viral loads on routine monitoring; and those who become unwell. CD4 testing, the preferred tool to diagnose advanced HIV disease, has declined to very low levels in many resource‐limited settings, particularly in sub‐Saharan Africa, and this partly explains the lack of data for a number of countries and population groups [63]. The decline in CD4 testing capacity has been linked to budget limitations, forcing programmes to prioritize viral load monitoring. A decrease in demand has resulted in reduced supply, with several manufacturers opting to withdraw their tests from the market [64]. Where CD4 count testing is unavailable, clinical staging is used to diagnose advanced HIV disease; however, staging has poor diagnostic accuracy, meaning that an important number of individuals who could benefit from interventions to reduce disease progression and mortality would be missed [65].

Until recently, advanced HIV disease was considered a challenge associated with late diagnosis and presentation to care at an advanced stage of illness [66]. As ART coverage has increased, there is a growing appreciation that a substantial number of people with advanced HIV disease are individuals who had started ART but subsequently disengaged from care, returning when they are ill [4]. This review further strengthens this finding that advanced HIV disease is common among both newly diagnosed, ART naïve individuals, and individuals who were previously diagnosed and have received treatment. Despite notable improvements in ART coverage during the time period chosen for this review, the relative estimated proportions of advanced HIV disease do not appear to have declined significantly. For studies that reported data across several years, no relationship was found between increased national ART coverage and advanced HIV disease prevalence [6, 24, 54]. For example, ART coverage in Thailand increased from 67% to 90%, but the proportion of individuals with advanced HIV disease remained constant throughout this period [6, 67]. There was insufficient data to explore the contribution made by disengagement in care, and this remains an important research question.

Access to healthcare services was disrupted during the COVID‐19 pandemic, leading to a decreased ability to detect and treat advanced HIV disease during this period. This was reported by several studies included in this review which compared the proportion of patients presenting with advanced HIV disease before and during the COVID‐19 pandemic. A study from Malawi found that hospital admissions halved in 2020 compared to historical data from 2017, attributing this change to difficulties in reaching healthcare facilities due to lockdown and fear of COVID‐19 infection [29]. Another study, from Uganda, reported that only 9% of people living with HIV presented with advanced HIV disease during COVID‐19 lockdowns (March−July 2020) compared to 16% during the period between July 2019 and February 2020 [43].

A recent study assessed the proportion of individuals with advanced HIV disease using data from population‐based HIV impact assessment household surveys and reported a prevalence of 10%, notably lower than the figure provided in this review [68]. This difference is explained by the fact that this review focused on individuals presenting to healthcare settings for whom a CD4 count was done and so more likely to have advanced HIV disease. This underscores the importance of considering setting when reporting estimates of advanced HIV disease.

### 4.1 Limitations of the available evidence

This review identified studies from a range of settings across all WHO regions and different economic areas, and included both published and unpublished data. The available evidence has several limitations. The majority of studies were from the African region, reflecting the global burden of HIV. There was little data from other regions, notably the eastern Mediterranean and Latin America, and few studies were identified among children; this highlights the need for data from a broader set of patient populations and countries. All children younger than 5 years are defined as having advanced disease except for those receiving ART for more than 1 year and who are clinically stable [69]; as such, the proportion of children with advanced HIV disease is likely to be higher than reported in this review. There were also fewer studies reporting data from inpatient settings.

It should also be emphasized that even among the included studies, data on CD4 cell count was for the most part limited: only a sub‐population of (possibly non‐random) people living with HIV who had a CD4 measured contributed data. For example, in IeDEA programmes, CD4 testing has become extremely limited in many settings [63], and people who do get a CD4 measurement may have higher levels of severe immune suppression compared to all people living with HIV starting ART in the country. Another limitation to note is that meta‐analyses of aggregate data are prone to ecological bias, and trends in CD4 cell count changes would be more reliably assessed using individual‐level data [70].

### 4.2 Limitations of the review methodology

This review used standard systematic review search methods, including multiple database and conference abstract screening; this information was further supported by data provided by IeDEA and from the national programme of Thailand. An important limitation of this review is the limited search strategy, including restricted search terms and limited database searches—this is why we choose to characterize this study conservatively as a rapid review. Studies that reported on the proportion of individuals with a CD4 <200 cells/mm^3^ but did not use the term advanced HIV disease, would not have been captured by this review. The use of such a focused search strategy was a pragmatic choice based on available resources, and the review was identified as a rapid review to indicate this limitation. We complemented this search through the inclusion of conference abstracts and unpublished data, including data provided by the IeDEA consortium, and national programme data. The identification of additional studies could strengthen some of the subgroup estimates where the available data was limited, in particular for children and for a number of countries. Nevertheless, the overall estimate was consistent across a number of subgroups and additional studies would be unlikely to change this estimate importantly.

## 5 CONCLUSIONS

This review provides further evidence of the continued high proportion of individuals who (re)present to care with advanced HIV disease. Future research should be directed to supporting a better understanding of reasons for both late diagnosis and disengagement in care as key drivers sustaining the high burden of advanced HIV disease. There is also a need for more research to better determine the outcomes of people diagnosed with advanced HIV disease, including the effectiveness of interventions to prevent, diagnose and treat opportunistic infections, and causes of severe disease, hospitalization and mortality. Diagnosing individuals with advanced HIV remains an important challenge to overcome, to be able to direct appropriate preventive, diagnostic and therapeutic interventions to prevent progression to severe illness and death. CD4 testing thus remains an essential tool in the programmatic response to advanced HIV disease.

## COMPETING INTERESTS

The authors declare no competing interests.

## AUTHORS’ CONTRIBUTIONS

Conceptualization: NF, DS and JJ. Data curation: NF, RK, DS and AR. Formal analysis: NF. Methodology: NF, RK, DS and JJ. Project administration: NF. Software: NF. Supervision: NF and AJ. Validation: NF and DS. Visualization: NF. Writing original draft: NF. Writing—review and editing: NF, RK, DS, JJ, OS, GP, MD and AR.

## FUNDING

This work was supported by a grant from the Gates Foundation (INV‐070‐0909) The International Epidemiology Databases to Evaluate AIDS (IeDEA) is supported by the U.S. National Institutes of Health's National Institute of Allergy and Infectious Diseases, the Eunice Kennedy Shriver National Institute of Child Health and Human Development, the National Cancer Institute, the National Institute of Mental Health, the National Institute on Drug Abuse, the National Heart, Lung, and Blood Institute, the National Institute on Alcohol Abuse and Alcoholism, the National Institute of Diabetes and Digestive and Kidney Diseases, and the Fogarty International Center: **Asia‐Pacific**, U01AI069907; **CCASAnet**, U01AI069923; **Central Africa**, U01AI096299; **East Africa**, U01AI069911; **NA‐ACCORD**, U01AI069918; **Southern Africa**, U01AI069924; **West Africa**, U01AI069919. Informatics resources are supported by the Harmonist project, R24AI24872.

## DISCLAIMER

This work is solely the responsibility of the authors and does not necessarily represent the official views of any of the institutions mentioned above.

## Supporting information



Supporting Information

## Data Availability

The data that support the findings of this study are openly available in the published articles included in this review and summarized in Table 1. Data from IeDEA are available upon reasonable request. Investigators wishing to work with IeDEA data should contact the regional data centres (see www.iedea.org) and submit a concept sheet for their intended analysis and data request.
