# Supplementary material for: Global prevalence of advanced HIV disease in healthcare settings: a rapid review
Source: J Int AIDS Soc. 2025 Feb 6;28(2):e26415. doi: 10.1002/jia2.26415 (PMC11802239; doi:10.1002/jia2.26415)
Supplement: Supplementary file 1 — Supporting Information [file JIA2-28-e26415-s001.docx]

**Risk of Bias assessment**

| **Study** | **Published** | **Representative sampling** | **Prospective data collection** | **All got CD4** | **ART status reported** | **CD4 method stated** |
| --- | --- | --- | --- | --- | --- | --- |
| Benzekri | Yes | Yes | Yes | Yes | Yes | No |
| Alli | No | No | Yes | Yes | No | No |
| Baldeh | Yes | No | Yes | Yes | Yes | Yes |
| Barak | Yes | No | Yes | No | Yes | No |
| Birhanu | Yes | Yes | Yes | Yes | Yes | Yes |
| Brennan | Yes | Yes | Yes | Yes | Yes | No |
| Bwalya | No | No | Yes | Yes | No | No |
| Camaci | Yes | No | Yes | Yes | Yes | No |
| Chabikuli | Yes | Yes | No | No | No | No |
| Dat | Yes | No | Yes | Yes | Yes | Yes |
| Ditondo | Yes | Yes | No | No | Yes | Yes |
| Elgalib | Yes | Yes | No | No | Yes | No |
| Garcia Ruiz De Morales | No | Yes | Yes | Yes | Yes | No |
| Gils | Yes | No | Yes | Yes | Yes | Yes |
| Giménez-Arufe | Yes | Yes | No | Yes | No | No |
| Glencross | Yes | Yes | Yes | Yes | No | No |
| Hamzah | No | No (hospital) | Yes | Yes | Yes | No |
| Hassan | Yes | Yes | No | No | Yes | No |
| Heller | Yes | No | No | No | Yes | Yes |
| Hu | Yes | Yes | No | No | Yes | Yes |
| Huang | No | Yes | No | Yes | Yes | No |
| IeDEA | Yes | No | Yes | No | Yes | No |
| Jiang | Yes | No | Yes | Yes | Yes | No |
| Kerschberger | Yes | Yes | Yes | Yes | Yes | No |
| Kumar | Yes | Yes | Yes | Yes | No | No |
| Lamp | Yes | Yes | No | Yes | No | Yes |
| Lauscher | No | Yes | No | Yes | Yes | No |
| Lebelonyane | Yes | Yes | Yes | Yes | Yes | Yes |
| Leeme | Yes | Yes | No | Yes | No | No |
| Levy-Braide | No | Yes | Yes | No | No | No |
| Li | Yes | No | No | No | No | No |
| Lifson | Yes | Yes | Yes | No | No | No |
| Lin | No | No (hospital) | Yes | Yes | Yes | No |
| Mambetov | No | Yes | Yes | Yes | Yes | No |
| Masaba | Yes | Yes | No | No | Yes | No |
| Mihaja Raberahona | Yes | Yes | No | No | No | Yes |
| Mugenyi | Yes | Yes | No | No | Yes | No |
| Musengimana | Yes | Yes | No | No | Yes | No |
| Mwakisambwe | No | Yes | Yes | Yes | Yes | No |
| Nalintya | No | Yes | No | Unclear | No | No |
| Nalugoda | No | Yes | Yes | Yes | Yes | No |
| Nalugoda | No | Yes | Yes | Yes | Yes | Yes |
| Ncayiyana | No | Yes | Yes | Yes | Yes | No |
| Ndlovu | Yes | No (hospital) | Yes | Yes | No | Yes |
| Ngongo | Yes | Yes | No | No | Yes | No |
| Nhampossa | Yes | Yes | No | Yes | Yes | No |
| Noknoy | No | Yes | Yes | Yes | Yes | No |
| Oboho | Yes | Yes | Yes | Yes | Yes | Yes |
| Osler | Yes | Yes | Yes | Yes | Yes | No |
| Otani | No | Yes | Yes | Yes | Yes | No |
| Ousley | Yes | No | Yes |  | Yes | Yes |
| Owachi | Yes | No | No | No | Yes | No |
| Parisi | No | Yes | Yes | Yes | Yes | No |
| Pedrola | No | Yes | Yes | Yes | Yes | Yes |
| Samayoa | No | Yes | Yes | No | Yes | No |
| Shi | No | Yes | No | Yes | Yes | No |
| Sornillo | No | Yes | Yes | No | Yes | No |
| Spinelli | No | Yes | Yes | Yes | No | No |
| Ssempijja | No | Yes | Yes | Yes | Yes | No |
| Stelzle | No | Yes | Yes | Yes | Yes | No |
| Stoger | Yes | No | Yes | No | Yes | No |
| Subramanian | No | Yes | Yes | Yes | No | No |
| Tegegne | No | No | No | Yes | Yes | No |
| Tiam | Yes | Yes | Yes | Yes | Yes | No |
| Yendewa | Yes | No | No | No | Yes | Yes |

ART, antiretroviral therapy

**GRADE Table**

| **Outcome** | **Number/ type of studies** | **Risk of bias** | **Consistency** | **Precision** | **Directness** | **Certainty of the evidence** | **Main findings** |
| --- | --- | --- | --- | --- | --- | --- | --- |
| % AHD | 119 observational studies | Moderate | Some Inconsistency | Some imprecision | No indirectness | Low | Inpatient settings 44.3%  (95%CI 39.1-49.6%)  Outpatient settings 33.5%  (95%CI 31.5-35.4%) |

AHD, advanced HIV disease
